# Cardiovascular Risk Factors as Predictors of Nutritional Status in Older Mexican Adults

**DOI:** 10.3390/nu16162662

**Published:** 2024-08-12

**Authors:** Dennys Alexandra Hernández-Torres, Myrna Elizabeth López-Hernández, Maria Elena Camacho-Moll, Mario Bermúdez de León, Katia Peñuelas-Urquides, Laura Adiene González-Escalante, Román González-Reyna, Darinka Laillete García-Leija, Brenda Leticia Escobedo-Guajardo

**Affiliations:** 1 Unidad De Medicina Familiar No. 32, Instituto Mexicano del Seguro Social, Guadalupe C.P. 67140, Nuevo León, Mexico; denya882502@gmail.com (D.A.H.-T.); myrna.lopezh@imss.gob.mx (M.E.L.-H.); roman.gonzalez@imss.gob.mx (R.G.-R.); darinka.garcia@imss.gob.mx (D.L.G.-L.); 2 Escuela de Medicina, Universidad de Monterrey, San Pedro Garza García C.P. 66238, Nuevo León, Mexico; maria.camachomo@imss.gob.mx (M.E.C.-M.); mario.bermudez@imss.gob.mx (M.B.d.L.); 3 Departamento de Biología Molecular, Centro de Investigación Biomédica del Noreste, Instituto Mexicano del Seguro Social, Monterrey C.P. 64720, Nuevo León, Mexico; katia.penuelasu@imss.gob.mx (K.P.-U.); laura.gonzaleze@imss.gob.mx (L.A.G.-E.)

**Keywords:** nutritional status, malnutrition, heart disease risk factors, aging

## Abstract

Aging is commonly accompanied by increased cardiovascular risk and diet plays a crucial role in health condition. The aim of this study was to determine cardiovascular risk factors as predictors of nutritional risk in Mexican older adults. A cross-sectional study on Mexican patients aged ≥60 years with cardiovascular risk factors affiliated with a medical unit in Northeast Mexico was performed from July to December 2021. The nutritional risk evaluations were performed using the Mini Nutritional Assessment (MNA) questionnaire. After a multivariate analysis, the cardiovascular risk factors identified as independent predictors of risk of malnutrition were hypertriglyceridemia (adjusted OR (AOR): 1.8; 95% CI: 1.03–3.14; *p* = 0.04) and systolic hypertension I (AOR: 2.28; 95% CI: 1.04–5.02; *p* = 0.041); age over 80 years (AOR: 5.17; 95% CI: 1.83–14.65, *p* = 0.002) and elementary school education (AOR: 2.34; 95% CI: 1.20–4.55; *p* = 0.013) were also related. The cross-sectional design and single-center approach of this study limits the generalizability of the results; however, conducting timely evaluations of blood pressure, triglyceride levels, and risk of malnutrition using the MNA tool for patients aged ≥60 years could prevent illness and reduce mortality within this population group.

## 1. Introduction

In 2020, the World Health Organization and the United Nations designated the period 2021–2030 as the “decade of healthy aging”. The primary objective of this initiative is to promote activities and interventions that enhance the quality of life for older individuals [1]. Aging is commonly accompanied by the presence of comorbidities, with cardiovascular risk factors such as hypertension, diabetes, obesity, dyslipidemia, and tobacco smoking being the most prevalent [2]; diet plays a crucial role in the prevention of cardiovascular disease (CVD), modifying these risk factors [3]. Research focusing on older adults is necessary, given that the risk of malnutrition increases naturally due to changes in body composition, the deterioration of organ function, the presence of comorbidities, and the occurrence of geriatric syndromes with variable prevalence rates [4,5,6,7,8,9,10]. The selection of an appropriate nutrition assessment tool is indeed crucial, as it can greatly impact the accuracy of the diagnosis and subsequent treatment decisions. Several factors should be considered, including its reliability, its applicability to the target population, the information it provides, and its suitability for the care setting. Other aspects such as filling time and ease of use are important in clinical practice [11,12,13,14]. The Mini Nutritional Assessment (MNA) tool has been designed for use in older populations [15,16,17,18]. This instrument comprises 18 items, grouped in four dimensions including anthropometric, risk situations, diet survey, and health self-perception. The maximum score is 30 and the cut-off point is above 23.5. Values below 23.5 are considered as risk of malnutrition/malnutrition [19]. The long MNA assessment typically takes around 15 min to complete and has been extensively validated and readily available [13]. In 2020, Fuentes-Pimentel and Camacho-Guerrero reported 25.34% malnutrition and 49% at risk of malnutrition using the MNA questionnaire in a population of older adults affiliated with a family medicine unit in the city of León, Guanajuato, Mexico [10]. On the other hand, Aleman-Mateo et al. (2007) evaluated the nutritional status of non-institutionalized Mexican older adults with cardiovascular risk factors using serum albumin levels and anthropometry, reporting malnutrition values of 15.3% [20]. In both studies, high prevalences of overweight and obesity were identified; however, predictors of malnutrition were not reported. Variants of the MNA questionnaire have been applied in the Mexican elderly population. A short version of the MNA questionnaire (MNA-SF) was applied to hospitalized Mexican older adults, reporting a prevalence of 76.6% of malnutrition in a population with a mean age of around 75 years [21]. In addition, a modified version of the MNA has been suggested to assess the risk of malnutrition among older Mexican adults in the Mexican Health and Aging Study (MHAS) with data from the 2012, 2015, and 2018 waves of the MHAS, a nationally representative study of Mexicans aged 50 and older. The sample included 13,338 participants [17]. A recent comparison between several tools used to assess the nutritional status of older adults reported the MNA as one of the best in terms of its validation parameters [14,22].

The MNA scale has proven useful for identifying risk of malnutrition, proving to be even more effective than assessing serum albumin levels alone [20]; furthermore, it has shown predictive value in relation to outcomes such as mortality and emergency room discharge in older adults [15]. In Mexico, people aged 60 years and over are considered as older adults [23], and to our best knowledge, there are no studies focused on this population assessing the nutritional status with the MNA tool and its association with cardiovascular risk factors. Our study identifies traditional cardiovascular risk factors as independent predictors of malnutrition for Mexican older adults affiliated with health services of the Mexican Social Security Institute (IMSS). According to a national health and nutrition survey [24], in the past 10 years, there has been an increase in three of the most prevalent conditions among older adults, which are high blood pressure, diabetes, and hypercholesterolemia with 0.8%, 2.4%, and 5.1% increases, respectively, mainly associated with women, people over 80 years of age, and people from rural areas [24]. On the other hand, it is also reported that overweight and obesity are present in 40.5% and 34.0% of older adults, respectively, and that they tend towards normal weights as age increases [24]. Interestingly, previous studies have indicated that the predictive value of these traditional cardiovascular risk factors changes with age, demonstrating a U-shaped relationship with survival outcomes, with malnutrition, inflammation, comorbidities, and frailty as the possible underlying mechanisms [2]. The assessment of cardiovascular risk factors in older adults affiliated with the IMSS is a routine practice in the medical consultation, but nutritional assessment is not, even when the average BMI for this population places them within the overweight category [24]. The novelty of our study lies in the use of cardiovascular risk factors widely monitored in institutionalized older adults as predictors of poor nutritional status, suggesting a nutritional evaluation by the nutrition specialist using the MNA questionnaire, allowing the detection of cases of nutritional risk even before they imply an evident weight loss.

## 2. Methods

### 2.1. Participants

The present study is an analytical cross-sectional investigation carried out at a family medical unit of the IMSS, located in the municipality of Guadalupe, Nuevo Leon, in Northeast Mexico. This study included participants aged ≥60 years with cardiovascular risk factors. These participants attended the medical unit for healthcare services from July to December 2021. This study was conducted according to the guidelines of the Declaration of Helsinki and was approved by the clinical research and ethics committees of the Mexican Social Security Institute with the registration number 2021-1909-116. Informed consent was obtained from participants according to guidelines in research with human subjects.

### 2.2. Selection Criteria

The inclusion criteria for this study included individuals of both sexes aged ≥60 years with one or more of the following cardiovascular risk factors: family history of CVD, hypertension, diabetes, obesity, dyslipidemia, and tobacco smoking. On the other hand, the exclusion criteria were a history of cerebrovascular disease accompanied by motor impairment, history of acute myocardial infarction, cardiac insufficiency or arrhythmia, cognitive impairment, malignancy, functional dependency, and those who declined to participate in this study.

### 2.3. Variables

A standardized questionnaire with sociodemographic items was carried out by a previously trained resident physician. The age of the participants was stratified into three categories: 60 to 69, 70 to 79, and 80 or above. Marital status was assessed as either (1) single or (2) in a relationship/married. Educational status was evaluated as either (1) elementary or no education or (2) middle/high school or higher. The history of cardiovascular risk factors was obtained through interviews and by reviewing clinical records from the three months preceding this study. The categories and reference values for these risk factors were determined based on the official regulatory organizations (Table 1). Anthropometric measurements were taken while participants wore light clothing and were barefoot, using calibrated instruments. The body mass index (BMI) was calculated by dividing weight (kg) by height (m^2^). The categories for BMI were based on parameters set by the WHO [25,26], classifying individuals as obese, overweight, healthy weight, or underweight. For a further analysis, the last two categories were combined. Systolic blood pressure (SBP) was categorized as follows: (1) normal, (2) elevated, (3) hypertension stage 1, and (4) hypertension stage 2. And diastolic blood pressure (DBP) was classified as (1) normal and (2) hypertension (including hypertension stages 1 and 2) (>130/80 mm Hg were the cut-off points for hypertension in accordance with Mexican regulations (NOM-030-SSA2-2016, DOF—Diario Oficial de la Federación) and the guidelines of the American Heart Association) [27]. Glycemia was classified according to fasting plasma glucose (FPG) levels as (1) normal and (2) diabetes [28]. Dichotomic categories were used for hypercholesterolemia, hypertriglyceridemia, cigarette smoking, and family history of CVD, with responses classified as either yes or no based on reference values or previously reported categories [29,30].

### 2.4. Nutritional Evaluation

Risk of malnutrition was measured using the MNA, which involved several components. Anthropometric measurements, including BMI, brachial circumference, calf perimeter, and weight loss, were taken by the same resident physician previously trained and assigned to the evaluation of all participants. To carry out this, the barefoot patient without heavy clothing was measured in height and weight with a calibrated scale with a stadiometer, Sentronik SE1500 (Scalemarket USA Corp, Miami, FL, USA). Calf circumference was measured with a flexible tape measure, in the region of maximum calf circumference, in centimeters. Brachial circumference was measured in a patient in an upright position, shoulders relaxed, arms at their sides, marking the midpoint of the distance between the tips of the acromion and the olecranon bones in centimeters. A global evaluation of lifestyle and diseases was conducted, where food intake parameters were assessed, including daily records of complete meals; dairy and meat consumption; intake of eggs, vegetables, and fruits; loss of appetite; water intake; and dependence on a feeder. The data collected covered a period of three months prior to this study. Patients were categorized based on their total MNA score, which ranged from 0 to 30. A score of 0 to 16 was classified as malnutrition; a score of 17 to 23.5 indicated risk of malnutrition; and a score of 24 to 30 corresponded to patients with normal nutritional status.

### 2.5. Statistical Analysis

The minimum sample size was calculated for a prevalence of 15% of malnutrition [20], a confidence level of 95%, and a margin of error of 5%. Demographic characteristics, cardiovascular risk factors, and related variables were reported using the mean and standard deviation (SD) or median and interquartile range, and frequency and percentages as appropriate. Categorical variables were analyzed using the chi-square or Mann–Whitney U test. Effect size was evaluated by adjusted odd ratios (AORs), using multivariate logistic regression models, which included the independent variables of sex, age, educational and marital status, BMI categories, family history of CVD, SBP, DBP, hypertriglyceridemia, total cholesterol, and FPG. If necessary, categories with fewer than five cases were regrouped for a logistic regression analysis to ensure statistical validity. The outcome variables for the nutritional status were classified into two groups: the nutritional risk group, which included individuals at risk of malnutrition or diagnosed as malnourished according to the MNA scores, and the normal nutrition group. Multicollinearity and goodness-of-fit for the regression model were evaluated, calculating the variance inflation factor (VIF) for the independent variables included in the model and with the Hosmer–Lemeshow test, respectively. Data were analyzed using the real statistics package for Microsoft Excel 365 MSO version 2404 (Microsoft, Redmond, Washington, DC, USA) and SPSS version 26 (International Business Machines Corporation, Armonk, New York, NY, USA), for a level of significance α of 0.05.

## 3. Results

### 3.1. Characteristics of Participants

A total of 311 older adults with cardiovascular risk factors were included in this study. This was higher compared to the minimum sample size of 194 participants calculated. The mean age of the participants was 69 ± 6.9 years (median: 68; interquartile range: 10), and 61.4% of them were women. Regarding educational status, 77.2% had elementary or no education, and 83.9% were living with a partner. The average BMI was 30.0 ± 5.1 kg/m^2^. SBP and DBP were 122.1 ± 10.3 and 76.7 ± 6.8 mm Hg, respectively. The FPG mean was 126.9 ± 45.4 mg/dL, and the total cholesterol mean was 188.9 ± 45.4 mg/dL, both around the limit values. The average triglyceride level was 194.3 ± 135.2 mg/dL. Among the cardiovascular risk factors, the most common was a family history of CVD (86.2%), followed by DBP hypertension (61.1%), hypertriglyceridemia (60.1%), hypercholesterolemia (47.9%), overweight (44.4%), obesity (43.4%), diabetes (38.3%), and SBP hypertension (29.0%). Only 1.6% of participants reported being cigarette smokers. Most participants (87.8%) had between three and six cardiovascular risk factors, and only 6.4% had a history of COVID-19. Significant differences were observed among groups for age, BMI, and MNA score (Table 2).

### 3.2. Nutritional Assessment

According to the MNA questionnaire, 65.3% (*n* = 203) were classified in the normal nutrition group, whereas 34.7% fell into the nutritional risk group, including those at risk of malnutrition (*n* = 103, 33.1%) and those diagnosed as malnourished (*n* = 5, 1.6%). Table 3 shows the distribution of participants by item by nutritional group. There were significative differences between groups except for items E, I related to neurological and physical diseases, and L about the intake of fruits and vegetables.

### 3.3. Bivariate Analysis

Only age and educational status had a significant association with nutritional risk in the bivariate analysis (*p* < 0.05) (Table 4).

**Figure 1:**
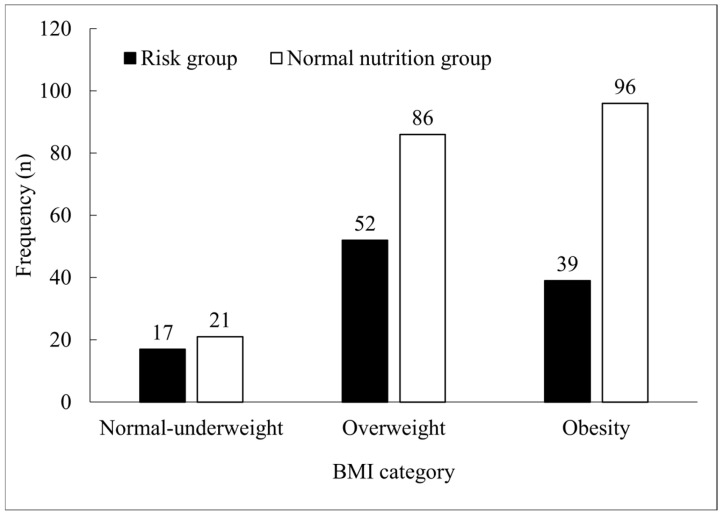
Comparison among groups according to BMI for each MNA nutritional status category. Obesity is prevalent in the normal nutritional group; meanwhile, overweight is predominant in the risk group. The risk group is composed of people with malnutrition and risk of malnutrition diagnoses. χ^2^, chi-square test, *p* = 0.120.

The distribution of BMI categories according to nutritional status is shown in Figure 1. In the nutritional risk group, overweight was the most prevalent category, with a prevalence of 48.1%. In contrast, the normal nutrition group had an obesity prevalence of 47.3%. However, no significant difference was observed between the groups (χ^2^ = 4.2; *p* = 0.120).

### 3.4. Multivariate Analysis

Categorical variables such as sex, age, educational and marital status, BMI, family history of CVD, SBP, DBP, FPG, total cholesterol, and hypertriglyceridemia were analyzed using multivariate logistic regression models. The goodness-of-fit of the regression model was evaluated with data from the Hosmer–Lemeshow test (chi-square = 8.332; *p* = 0.402); the absence of multicollinearity was verified by calculating the variance inflation factor (VIF) for the independent variables included in the model, obtaining values less than 2. The analysis revealed that being ≥80 years of age (AOR: 5.17; 95% CI: 1.83–14.65), having elementary or no education (AOR: 2.33; 95% CI: 1.20–4.55), having SBP values indicating hypertension stage 1 (AOR: 2.28; 95% CI: 1.04–5.02), and having hypertriglyceridemia (AOR: 1.80; 95% CI: 1.03–3.14) were predictors for the risk group (Table 5). Sex, family history of CVD, FPG, DBP, total cholesterol, cigarette smoking, and history of COVID-19 were not associated with the nutritional risk group.

## 4. Discussion

### 4.1. Nutritional Assessment

MNA is considered a simple tool to evaluate nutritional risks, more than other sophisticated tools not feasible for use at a healthcare unit that require validation for each ethnicity, and have been reported with some inconsistencies [7,12,31]. Since obesity and overweight are highly prevalent in the Mexican population [32] and the sample size of our study was limited, the selection of the MNA questionnaire minimizes phenotypic variability bias by identifying people at risk of malnutrition even before there are severe changes in weight or albumin levels [33]. This is not possible when using BMI alone as an indicator of nutritional status; it may result in underestimating the risk of malnutrition in older adults [20]. The MNA tool takes into account multiple aspects to identify risk of malnutrition beyond BMI alone [34] and for the assessment in older adults with cardiovascular disease risk factors like obesity and overweight, among others [34,35]. No information regarding malnutrition (using MNA tool) and cardiovascular risk factors in Mexican older adults has been reported. Also, MNA has demonstrated a good validity in sensitivity and predictive values compared with other tools designed for older adults [12,22,36].

In our study, the MNA questionnaire reported that 33.1% of participants were at risk of malnutrition. This prevalence is close to the 32.8% reported by a Mexican study conducted in 2020, which included 3218 older adults [4], supporting the validity of our analysis. However, other studies performed between 2016 and 2020 in Mexico have reported higher prevalence rates, ranging from 49.0% to 64.6% [6,8,10], all of them including a sample size similar to our study. On a global scale, risk of malnutrition among older adults has been reported to range from 32% to 76% [5,7].

A malnourished diagnosis in our study was found to be relatively low at 1.4%, compared to previous reports ranging from 4.1% to 25.34% [6,10]. Ávila et al. (2021) reported a lower risk of malnutrition in those affiliated with the IMSS compared to Seguro Popular. Both are public health services, but while the IMSS represents the working class of various economic strata, Seguro Popular represents people who do not have a formal labor affiliation [17]. On the other hand, one of the reasons for this discrepancy could be the study design and settings, as these factors have been previously mentioned in systematic reviews and meta-analyses where the prevalence of malnutrition differed significantly across the healthcare settings considered such as the community, outpatients, home-care services, and hospital, to mention some [35].

### 4.2. Demographic Variables and Risk of Malnutrition

Older age and lower educational level were associated with nutritional risk. Having no education or only elementary education were twice as likely to be in the nutritional risk group, which agrees with previous reports [6,37]. Participants aged ≥80 years had four times more probability of nutritional risk compared to those aged 60–69 years. Similar findings have been reported in other studies, attributing this association to geriatric syndromes and chronic diseases that are more prevalent in older groups, particularly due to factors such as motor impairment and dependency [38,39,40,41]. Nonetheless, there is a wide confidence interval reported for the age variable ≥80 years in our study, maybe because out of 27 participants in this category, only 9 of them (33.3%) were classified in the subgroup with normal nutrition. Therefore, results should be taken with caution. No significant association between sex and marital status and nutritional risk was observed in our study.

### 4.3. Cardiovascular Risk Factors and Risk of Malnutrition

In addition to demographic variables, cardiovascular risk factors have been previously evaluated. In Mexico, the burden of CVD has been increasing since 2007, primarily due to the aging population [42]. In a study by Dávila-Cervantes (2020), several cardiovascular risk factors were identified as prevalent in the Mexican population, including high SBP, dietary risks, high low-density lipoprotein (LDL) cholesterol, high BMI, and high FPG levels, all of which were associated with older age [42]. In our study, we found that among the main traditional cardiovascular risk factors, only SBP (hypertension stage 1) and hypertriglyceridemia were significantly associated with nutritional risk. It should be noted that no association with nutritional status was found when the number of cardiovascular risk factors in patients was analyzed, as shown in previous reports [43].

Hypertension was the most prevalent condition among the cardiovascular risk factors in our study, with a higher prevalence of diastolic hypertension (61.1%), and systolic hypertension I significantly associated with malnutrition risk (Table 5) (cut-off point for hypertension according with Mexican regulations (NOM-030-SSA2-2016) and the guidelines of the American Heart Association) [27]. The prevalence of malnutrition in elderly patients with hypertension has been studied before using the MNA tool where a 36.7% risk of malnutrition was reported [43]. Furthermore, Merad-Boudia et al. (2016) demonstrated that the risk of malnutrition was three times higher in patients with hypertension who were under a treatment with three drugs per day compared to those who were not following a daily treatment or were treated with less than three drugs a day (75.8% vs. 24.2%) [43]. This agrees with our study where we demonstrate a greater risk of malnutrition and malnourishment in patients taking more than three prescription drugs per day (Table 3H).

In contrast to negative outcomes associated with hypertension, Abdelhafiz et al. (2012) reported that SBP levels between 110 and 140 mm Hg have been related to a decreased risk of cardiovascular events, even in the presence of diabetes and other chronic diseases; however, this finding is more relevant for adults aged 85 years and above [2].

In addition to the above, we observed a significant association between hypertriglyceridemia and the risk of malnutrition. Previous reports found no significant association between triglycerides and nutritional status even when there was a clear interaction between malnutrition and serum lipid profiles [16,44]. It is worth noting that overweight and obesity, which are conditions of malnutrition characterized by an excess of lipids, often coexist with micronutrient deficiencies in important vitamins and minerals [25]. A recent study in China found that vitamin B (B1, B2, B6, and B9) was negatively associated with obesity in Chinese middle-aged and older adults [45]. Furthermore, it has been shown that patients with morbid obesity under evaluation for bariatric surgery are found to have a high prevalence of nutritional deficiencies [46,47,48]. Malnutrition in obese patients has also been reported in other studies [49].

Overweight and obesity were prevalent among the participants in our study. Overweight does not necessarily mean well nourished. A study performed in Turkey in a total of 187 elderly patients with overweight or obesity using the MNA tool demonstrated a >25 BMI prevalence of 49.7% in the elderly patients [50]. In our population, we confirm the presence of malnutrition in patients with overweight or obesity where the prevalence of nutritional risk in patients with overweight was 48.1%, whereas for obesity, it was 47.3% (Table 5).

The MNA tool takes into consideration other measurements besides BMI to determine nutritional status (brachial circumference, calf perimeter, and weight loss; a global evaluation of lifestyle and diseases was conducted, and food intake parameters were assessed, including diary records of complete meals; meat consumption; intake of eggs, vegetables, and fruits; loss of appetite; water intake; and dependence on a feeder). Previously, a BMI of ≥25 was identified as a common factor contributing to metabolic abnormalities [20]. More recently, it has been shown that BMI values below 23 kg/m^2^ and above 33 kg/m^2^ are associated with an increased risk of mortality in the older population [51]; nonetheless, overweight and obesity are linked to better functional status [34]. Therefore, it is possible to have a BMI of more than 25 along with being well nourished. Having said that, BMI was not statistically significant in the nutritional risk group.

However, it is important to note that the association between risk of malnutrition and overweight or obesity does not always predict negative outcomes. Chen et al. (2022) evaluated a group of individuals with a median age of 62 ± 1.2 years (*n* = 239) who had malnutrition–sarcopenia associated with obesity [52]. It was found that the malnutrition–sarcopenia syndrome influenced cardiovascular mortality in the non-obese subgroup; however, it did not reach statistical significance in obese participants [52]. Furthermore, Wleklik et al. (2018) reviewed the role of the nutritional status in elderly patients with heart failure and mentioned that evidence indicates that malnutrition frequently co-occurs with chronic heart failure [53] with obese and overweight patients at a higher risk to develop heart failure than those with normal weight; however, when only studying patients with heart failure, obesity and overweight were protecting factors for death (3), acting as the “elderly paradox” cited by Abdelhafiz et al. (2012), referring to a reverse metabolism with cardiovascular risk factors becoming protective factors [2]. Compared to our results, we did not observe a significant difference in malnutrition risk in obese nor overweight patients (Table 5).

### 4.4. Limitations and Strengths

This study had certain limitations, including the lack of older adults without CVD during the data collection period for comparison. The sample size was insufficient for the subgroup analysis of the malnourished diagnosis. Factors such as physical activity and alcohol consumption were not considered. The data obtained with the MNA survey can be affected by an external bias caused by social desirability or approval, especially in this case where anonymity and confidentiality cannot be guaranteed at the time of data collection. The cross-sectional design, since it only represents a one-time measurement of both the alleged predictor and effect, is useful for establishing preliminary evidence in planning a future advanced study, but it has as a limitation the directionality of the relationship found [54]. However, for the purposes of this study, the variables identified as predictors of malnutrition are proposed as a starting point for the evaluation of nutritional status and to identify a probable condition of malnutrition in Mexican older adults affiliated with the IMSS. Finally, the single-center approach of this study limits the generalizability of the results to other healthcare and community settings, as has been demonstrated by Cereda et al. (2016), in a systematic review and meta-analysis of prevalence data using MNA [35].

This study had strengths in terms of data collection methods. The information on CVD was obtained from clinical records rather than relying solely on self-reports by the participants. This approach helped to eliminate the potential bias of participants incorrectly associating controlled or uncontrolled cardiovascular risk factors with their malnutrition status at the time of the interview. Although this study was descriptive in nature, it provided an overview of the characteristics of the population under study.

## 5. Conclusions

Our results confirm cardiovascular risk factors hypertriglyceridemia and hypertension as predictors of the risk of malnutrition. A low educational level and age over 80 years were associated demographic factors. The prevention of nutritional diseases remains key to reducing the mortality risk associated with older age and related geriatric syndromes, especially in an era where the older population is expected to become predominant. Furthermore, this study proposes the need for future research to conduct causality studies that can confirm the inferences and relationships related to the variable being investigated.

## Figures and Tables

**Table 1:** Categories and reference values for variables.

Variables	Categories	Reference Values
Body mass index [25,26] ^†^	Underweight	<18.5 kg/m^2^
	Normal weight	18.5–24.9 kg/m^2^
	Overweight	25–29 kg/m^2^
	Obesity	≥30 kg/m^2^
Systolic blood pressure [27]	Normal	<120 mm Hg
	Elevated	120–129 mm Hg
	Hypertension stage 1	130–139 mm Hg
	Hypertension stage 2	≥140 mm Hg
Diastolic blood pressure [27] ^‡^	Normal	<80 mm Hg
	Hypertension stage 1	130–139 mm Hg
	Hypertension stage 2	≥140 mm Hg
Fasting plasma glucose [28]	Normal	<126 mg/dL
	Diabetes	≥126 mg/dL
Total cholesterol [29]	Normal	<190 mg/dL
	Raised	≥190 mg/dL
Triglycerides [30] ^§^	Normal	<150 mg/dL
	Moderate	150–499 mg/dL
	Severe	≥500 mg/dL

^†^ Categories for analysis are underweight, normal weight, overweight, and obesity. ^‡^ Categories for analysis are normal and hypertension (including stage 1 and stage 2 categories). ^§^ Categories for analysis are normal and hypertriglyceridemia (moderate and severe categories).

**Table 2:** Clinical data comparison between groups.

Variable	Total (*n* = 311) Mean (SD)	Risk of Malnutrition (*n* = 108) Mean (SD)	Normal Nutrition (*n* = 203) Mean (SD)	*p* Value
Age (years)	69.3 (±6.9)	70.68 (±7.8)	68.5 (±6.2)	0.029 *
BMI (kg/m^2^)	30.0 (±5.1)	29.4 (±5.8)	30.3 (±4.7)	0.039 *
SBP (mm Hg)	122.1 (±10.3)	123.4 (±10.3)	121.4 (±10.3)	0.070
DBP (mm Hg)	76.7 (±6.8)	77.0 (±6.9)	76.5 (±6.7)	0.245
FPG (mg/dL)	126.9 (±45.4)	126.9 (±47.2)	126.9 (±44.5)	0.984
Total cholesterol (mg/dL)	188.9 (±45.4)	190.7 (±47.6)	188.0 (±44.2)	0.904
Triglycerides (mg/dL)	194.3 (±135.3)	214.6 (±173.3)	183.5 (±108.8)	0.125
MNA score	24.1 (±2.2)	21.78 (±2.0)	25.3 (±1.1)	<0.001 ***

* *p* < 0.05, *** *p* < 0.001, Mann—Whitney U test. BMI, body mass index; DBP, diastolic blood pressure; FPG, fasting plasma glucose; MNA, Mini Nutritional Assessment; SBP, systolic blood pressure.

**Table 3:** Results of the Mini Nutritional Assessment questionnaire in Mexican older adults with cardiovascular risk factors (*n* = 311).

Item		Value	Normal Nutritional Status (*n* = 203)	Risk of Malnutrition (*n* = 108)	*p* Value
			*n* (%)	*n* (%)	
A.	Has food intake declined over the past 3 months due to loss of appetite, digestive problems, chewing or swallowing difficulties?				
	Severe decrease in food intake	0	-	1 (1)	<0.001 *
	Moderate decrease in food intake	1	32 (15.8)	55 (53.4)	
	No decrease in food intake	2	171 (84.2)	52 (50.5)	
B.	Weight loss during the past 3 months				
	Weight loss greater than 3 kg (6.6 lbs)	0	1 (0.5)	1 (1)	<0.001 *
	Does not know	1	15 (7.4)	40 (38.8)	
	Weight loss between 1 and 3 kg (2.2 and 6.6 lbs)	2	10 (4.9)	18 (17.5)	
	No weight loss	3	177 (87.2)	49 (47.6)	
C.	Mobility				
	Bed- or chair-bound	0	-	5 (4.9)	<0.001 *
	Able to get out of bed/chair but does not go out	1	10 (4.9)	25 (24.3)	
	Goes out	2	193 (95.1)	78 (75.7)	
D.	Has suffered psychological stress or acute disease in the past 3 months?				
	Yes	0	2 (1)	23 (22.3)	<0.001 ^a,^*
	No	2	201 (99)	85 (82.5)	
E.	Neuropsychological problems				
	Severe dementia or depression	0	-	2 (1.9)	0.120 ^a^
	Mild dementia	1	-	-	
	No psychological problems	2	203 (100)	106 (102.9)	
F.	Body mass index (BMI) = weight in kg/(height in m^2^)				
	BMI less than 19	0	-	5 (4.9)	<0.001 *
	BMI of 19 to less than 21	1	1 (0.5)	1 (1)	
	BMI of 21 to less than 23	2	1 (0.5)	6 (5.8)	
	BMI of 23 or greater	3	201 (99)	96 (93.2)	
G.	Lives independently (not in nursing home or hospital)				
	Yes	1	174 (85.7)	75 (72.8)	0.001 ^a,^*
	No	0	29 (14.3)	33 (32)	
H.	Takes more than 3 prescription drugs per day				
	Yes	0	163 (80.3)	98 (95.1)	0.022 ^a^
	No	1	40 (19.7)	10 (9.7)	
I.	Pressure sores or skin ulcers				
	Yes	0	2 (1)	4 (3.9)	0.188 ^a^
	No	1	201 (99)	104 (101)	
J.	How many full meals does the patient eat daily?				
	1 meal	0	-	-	
	2 meals	1	114 (56.2)	74 (71.8)	0.039 ^a,^*
	3 meals	2	89 (43.8)	34 (33)	
K.	Selected consumption markers for protein intake At least one serving of dairy products (milk, cheese, yoghurt) per day Two or more servings of legumes or eggs per week Meat, fish, or poultry every day				
	If 0 or 1, yes	0	17 (8.4)	22 (21.4)	0.010 *
	If 2, yes	0.5	175 (86.2)	81 (78.6)	
	If 3, yes	1	11 (5.4)	5 (4.9)	
L.	Consumes two or more servings of fruit or vegetables per day?				
	No	0	118 (58.1)	61 (59.2)	0.810
	Yes	1	85 (41.9)	47 (45.6)	
M.	How much fluid (water, juice, coffee, tea, milk…) is consumed per day?				
	Less than 3 cups	0	77 (37.9)	60 (58.3)	0.008 *
	3 to 5 cups	0.5	124 (61.1)	48 (46.6)	
	More than 5 cups	1	2 (1)	-	
N.	Mode of feeding				
	Unable to eat without assistance	0	-	-	0.003 *
	Self-fed with some difficulty	1	1 (0.5)	7 (6.8)	
	Self-fed without any problem	2	202 (99.5)	101 (98.1)	
O.	Self-view of nutritional status				
	Views self as being malnourished	0	-	-	<0.001 *^,a^
	Is uncertain of nutritional state	1	8 (3.9)	37 (35.9)	
	Views self as having no nutritional problem	2	195 (96.1)	71 (68.9)	
P.	In comparison with other people of the same age, how does the patient consider his/her health status?				
	Not as good	0	-	-	<0.001 *
	Does not know	0.5	88 (43.3)	87 (84.5)	
	As good	1	51 (25.1)	12 (11.7)	
	Better	2	64 (31.5)	9 (8.7)	
Q.	Mid-arm circumference (MAC) in cm				
	MAC less than 21	0	-	2 (1.9)	<0.001 *
	MAC at 21 to 22	0.5	2 (1)	17 (16.5)	
	MAC greater than 22	1	201 (99)	89 (86.4)	
R.	Calf circumference (CC) in cm				
	CC less than 31	0	-	10 (9.7)	<0.001 ^a,^*
	CC at 31 or greater	1	203 (100)	98 (95.1)	

Normal nutritional status: 24 to 30 points; risk of malnutrition group less than 23.5 points. * *p* < 0.05, Pearson’s chi-square test. ^a^ Fisher’s Exact test.

**Table 4:** Distribution of groups and bivariate association analysis.

Variables	Total *n* = 311	Risk of Malnutrition *n* = 108	Normal Nutrition *n* = 203	χ^2^ *p* Value
	*n* (%)	*n* (%)	*n* (%)	
Sex				
Women	191 (61.4)	62 (57.4)	129 (63.5)	0.290
Men	120 (38.6)	46 (42.6)	74 (36.5)	
Age				
60–69	175 (56.3)	56 (51.9)	119 (58.6)	0.001 *
70–79	109 (35.0)	34 (31.5)	75 (36.9)	
≥80	27 (8.7)	18 (16.7)	9 (4.4)	
Educational status				0.014 *
Middle/high school or higher	71 (22.8)	16 (14.8)	55 (27.1)	
Elementary/no education	240 (77.2)	92 (85.2)	148 (72.9)	
Marital status				
In a relationship/married	261 (83.9)	91 (84.3)	170 (83.7)	0.508
Single	50 (16.1)	17 (15.7)	33 (16.3)	
Family history of CVD				
No	43 (13.8)	11 (10.2)	32 (15.8)	0.175
Yes	268 (86.2)	97 (89.8)	171 (84.2)	
DBP				
Normal	121 (38.9)	36 (33.3)	85 (41.9)	0.141
Hypertension	190 (61.1)	72 (66.7)	118 (58.1)	
SBP				
Normal	68 (21.9)	20 (18.5)	48 (23.6)	0.138
Elevated	153 (49.2)	49 (45.4)	104 (51.2)	
Hypertension 1	64 (20.6)	30 (27.8)	34 (16.7)	
Hypertension 2	26 (8.4)	9 (8.3)	17 (8.4)	
Hypertriglyceridemia				
No	124 (39.9)	39 (36.1)	85 (41.9)	0.323
Yes	187 (60.1)	69 (63.9)	118 (58.1)	
Hypercholesterolemia				
No	162 (52.1)	55 (50.9)	107 (52.7)	0.673
Yes	149 (47.9)	53 (49.1)	96 (47.3)	
FPG				
Normal	192 (61.7)	67 (62.0)	125 (61.6)	0.937
Diabetes	119 (38.3)	41 (38.0)	78 (38.4)	
Cigarette smoking				
No	306 (98.4)	106 (98.1)	200 (98.5)	1.000 ^†^
Yes	5 (1.6)	2 (1.9)	3 (1.5)	
Number of risk factors				
<3	38 (12.2)	10 (9.3)	28 (13.8)	0.245
≥3	273 (87.8)	98 (90.7)	175 (86.2)	
History of COVID-19				
No	291 (93.6)	104 (96.3)	187 (92.1)	0.153
Yes	20 (6.4)	4 (3.7)	16 (7.9)	

* *p* < 0.05. χ^2^, chi-square test. ^†^ Fisher Exact test. DBP, diastolic blood pressure; FPG, fasting plasma glucose; SBP, systolic blood pressure.

**Table 5:** Multivariate regression analysis and adjusted odds ratios for nutritional risk group (*n* = 311).

	B	Standard Error	Wald	df	*p* Value	Exp(b)	95% CI
Sex							
Women						Ref	
Men	0.3	0.3	1.2	1	0.282	1.35	(0.78–2.35)
Age			11.3	2	0.004 *		
60–69						Ref	
70–79	−0.1	0.3	0.1	1	0.718	0.90	(0.51–1.59)
≥80	1.6	0.5	9.6	1	0.002 *	5.17	(1.83–14.65)
Educational status							
Middle/high school or higher						Ref	
Elementary/no education	0.8	0.3	6.2	1	0.013 *	2.34	(1.20–4.55)
Marital status							
In a relationship/married						Ref	
Single	−0.5	0.4	1.3	1	0.260	0.62	(0.27–1.43)
Family history of CVD							
No						Ref	
Yes	0.3	0.4	0.7	1	0.407	1.39	(0.64–3.03)
SBP			6.9	3	0.076		
Normal						Ref	
Elevated	0.0	0.3	0.0	1	0.925	1.03	(0.53–2.02)
Hypertension 1	0.8	0.4	4.2	1	0.041 *	2.28	(1.04–5.02)
Hypertension 2	0.0	0.5	0.0	1	0.930	0.95	(0.33–2.78)
DBP							
Normal						Ref	
Hypertension 1	0.3	0.3	1.3	1	0.263	1.36	(0.79–2.32)
Triglycerides							
Normal						Ref	
Hypertriglyceridemia	0.6	0.3	4.2	1	0.040 *	1.80	(1.03–3.14)
Total cholesterol							
Normal						Ref	
Raised	0.0	0.3	0.0	1	0.928	1.02	(0.60–1.74)
BMI			2.9	2	0.234		
Normal–underweight						Ref	
Overweight	−0.3	0.4	0.6	1	0.423	0.72	(0.32–1.61)
Obesity	−0.7	0.4	2.5	1	0.113	0.51	(0.22–1.17)
FPG							
Normal						Ref	
Diabetes	−0.3	0.3	1.1	1	0.301	0.75	(0.44–1.29)

B, *y* axis interjection; BMI, body mass index; CI, confidence interval; CVD, cardiovascular disease; DBP, diastolic blood pressure; df, degree freedom; Exp(b), multivariate odds ratio; FPG, fasting plasma glucose; Ref, reference; SBP, systolic blood pressure. * *p* < 0.05.

## Data Availability

The datasets generated and/or analyzed during the current study are available from the corresponding author on reasonable request.

## References

[1] United Nations, Population Division, Department of Economic and Social Affairs (2023). World Social Report 2023: Leaving No One Behind in an Ageing World. https://www.un.org/development/desa/pd/content/launch-world-social-report-2023.

[2] Abdelhafiz A.H., Loo B.E., Hensey N., Bailey C., Sinclair A. (2012). The U-shaped Relationship of Traditional Cardiovascular Risk Factors and Adverse Outcomes in Later Life. Aging Dis..

[3] Bernal Y., Candela Y., Salgado T. (2017). Vulnerabilidad alimentaria nutricional en el adulto mayor. Rev. Esp. Nutr. Comunitaria.

[4] Guzmán-Olea E., Agis-Juárez R.A., Bermúdez-Morales V.H., Torres-Poveda K., Madrid-Marina V., López-Romero D., Maya-Pérez E. (2020). Estado de salud y valoración gerontológica en adultos mayores mexicanos ante la pandemia por COVID-19. Gac. Médica México.

[5] Giraldo Giraldo N.A., Paredes Arturo Y.V., Idarraga Idarraga Y., Aguirre Acevedo D.C. (2017). Factores asociados a la desnutrición o al riesgo de desnutrición en adultos mayores de San Juan de Pasto, Colombia: Un estudio transversal. Rev. Española Nutr. Humana Dietética.

[6] Gonzalez-Vargas A., Gomez-Ortega M., Dimas-Altamirano B., Escalona-Franco M.E.V. (2016). Estado nutricional del adulto mayor, Almoloya de Juárez, Estado de México, 2015. Rev. Med. Investig..

[7] MacDonell S.O., Moyes S.A., Teh R., Dyall L., Kerse N., Wham C. (2023). Is the Utility of the GLIM Criteria Used to Diagnose Malnutrition Suitable for Bicultural Populations? Findings from Life and Living in Advanced Age Cohort Study in New Zealand (LiLACS NZ). J. Nutr. Health Aging.

[8] Bezares-Sarmiento V.R., León-González J.M., Pascacio-González M.R. (2017). Evaluación nutricional de población de adultos mayores de comunidades rurales de Chiapas. Rev. Esp. Nutr. Comunitaria.

[9] García S.M., Perri N., Leal M. (2017). Valoración de riesgo y vulnerabilidad nutricional y funcionabilidad de tejido músculo-esquelético, en adulto mayor internados en Sanatorios de la Trinidad durante los meses de febrero-marzo del año 2017. Rev. Esp. Nutr. Comunitaria.

[10] Fuentes-Pimentel L.E., Camacho-Guerrero A. (2020). Prevalencia del estado de desnutrición en los adultos mayores de la Unidad Médica Familiar Núm. 53 de León, Guanajuato, México. Residente.

[11] House M., Gwaltney C. (2022). Malnutrition screening and diagnosis tools: Implications for practice. Nutr. Clin. Pract..

[12] Charlton K.E., Kolbe-Alexander T.L., Nel J.H. (2007). The MNA, but not the DETERMINE, screening tool is a valid indicator of nutritional status in elderly Africans. Nutrition.

[13] Anthony P.S. (2008). Nutrition screening tools for hospitalized patients. Nutr. Clin. Pract..

[14] Saghafi-Asl M., Vaghef-Mehrabany E., Karamzad N., Daeiefarshbaf L., Kalejahi P., Asghari-Jafarabadi M. (2018). Geriatric nutritional risk index as a simple tool for assessment of malnutrition among geriatrics in Northwest of Iran: Comparison with mini nutritional assessment. Aging Clin. Exp. Res..

[15] Kang M.G., Choi J.Y., Yoo H.J., Park S.Y., Kim Y., Kim J.Y., Kim S.W., Kim C.H., Kim K.I. (2022). Impact of malnutrition evaluated by the mini nutritional assessment on the prognosis of acute hospitalized older adults. Front. Nutr..

[16] Keshavarz S., Hosseini S., Amin A., Bakshandeh H., Maleki M., Shahinfard A., Hosseini S., Heidarali M. (2017). Nutritional status assessment of the elderly patients with congestive heart failure by mini nutritional assessment test. Res. Cardiovasc. Med..

[17] Avila J.C., Samper-Ternent R., Wong R. (2021). Malnutrition Risk among Older Mexican Adults in the Mexican Health and Aging Study. Nutrients.

[18] Taboada B., Zarate S., Isa P., Boukadida C., Vazquez-Perez J.A., Munoz-Medina J.E., Ramírez-González J.E., Comas-García A., Grajales-Muñiz C., Rincón-Rubio A. (2021). Genetic Analysis of SARS-CoV-2 Variants in Mexico during the First Year of the COVID-19 Pandemic. Viruses.

[19] Salva Casanovas A. (2012). The mini nutritional assessment. Twenty years contributing to nutritional assessment. Rev. Esp. Geriatr. Gerontol..

[20] Aleman-Mateo H., Esparza-Romero J., Romero R.U., Garcia H.A., Perez Flores F.A., Ochoa Chacon B.V., Valencia M.E. (2008). Prevalence of malnutrition and associated metabolic risk factors for cardiovascular disease in older adults from Northwest Mexico. Arch. Gerontol. Geriatr..

[21] Olmos Gutiérrez E., Mendieta Zerón E., Hinojosa Juárez A., Rivero Navarro M.A. (2021). Evaluación del estado nutricional en población geriátrica mexicana hospitalizada por medio del Mini Nutritional Assessment. CIMEL Cienc. Investig. Med. Estud. Latinoam..

[22] Silva D.F.O., Lima S., Sena-Evangelista K.C.M., Marchioni D.M., Cobucci R.N., Andrade F.B. (2020). Nutritional Risk Screening Tools for Older Adults with COVID-19: A Systematic Review. Nutrients.

[23] Gobierno de México Instituto Nacional de las Personas Adultas Mayores. Acciones y Programas. Updated 19 September 2023. https://www.gob.mx/inapam/acciones-y-programas/tarjeta-inapam-conoce-los-requisitos-para-obtener-la-tarjeta-inapam?idiom=es.

[24] Salinas-Rodriguez A., De la Cruz-Gongora V., Manrique-Espinoza B. (2020). Health conditions, geriatric syndromes andnutritional status of older adults in Mexico. Salud Publica México.

[25] World Health Organization Obesity and Overweight Updated 9 June 2021. https://www.who.int/news-room/fact-sheets/detail/obesity-and-overweight.

[26] World Health Organization Body Mass Index (BMI). https://www.who.int/data/gho/data/themes/topics/topic-details/GHO/body-mass-index.

[27] Whelton P.K., Carey R.M., Aronow W.S., Casey D.E., Collins K.J., Dennison Himmelfarb C., DePalma S.M., Gidding S., Jamerson K. A., Jones D. W. (2018). 2017 ACC/AHA/AAPA/ABC/ACPM/AGS/APhA/ASH/ASPC/NMA/PCNA Guideline for the Prevention, Detection, Evaluation, and Management of High Blood Pressure in Adults: A Report of the American College of Cardiology/American Heart Association Task Force on Clinical Practice Guidelines. Hypertension.

[28] American Diabetes Association (2023). Diabetes Overview/Understandign A1C/Diagnosis. https://diabetes.org/diabetes/a1c/diagnosis.

[29] World Health Organization (2012). Report of the Formal Meeting of Member States to Conclude the Work on the Comprehensive Global Monitoring Framework, Including Indicators, and a Set of Voluntary Global Targets for the Prevention and Control of Noncommunicable Diseases. https://apps.who.int/gb/NCDs/pdf/A_NCD_2-en.pdf.

[30] Jacobsen A., Savji N., Blumenthal R.S., Martin S.S. (2019). Hypertriglyceridemia Management according to the 2018 AHA/ACC Guideline. https://www.acc.org/latest-in-cardiology/articles/2019/01/11/07/39/hypertriglyceridemia-management-according-to-the-2018-aha-acc-guideline.

[31] Shamlan G., Albreiki M., Almasoudi H.O., Alshehri L.A., Ghaith M.M., Alharthi A.S., Aleanizy F.S. (2024). Nutritional status of elderly patients previously ill with COVID-19: Assessment with nutritional risk screening 2002 (NRS-2002) and mini nutritional assessment (MNA-sf). J. Infect. Public Health.

[32] Center for Disease Control and Prevention Overweight and Obesity. 3 June 2022. https://www.cdc.gov/obesity/basics/adult-defining.html.

[33] Vellas B., Guigoz Y., Garry P.J., Nourhashemi F., Bennahum D., Lauque S., Albarede J.L. (1999). The Mini Nutritional Assessment (MNA) and its use in grading the nutritional state of elderly patients. Nutrition.

[34] Bahat G., Tufan F., Saka B., Akin S., Ozkaya H., Yucel N., Erten N., Karan M.A. (2012). Which body mass index (BMI) is better in the elderly for functional status?. Arch. Gerontol. Geriatr..

[35] Cereda E., Pedrolli C., Klersy C., Bonardi C., Quarleri L., Cappello S., Turri A., Rondanelli M., Caccialanza R. (2016). Nutritional status in older persons according to healthcare setting: A systematic review and meta-analysis of prevalence data using MNA((R)). Clin. Nutr..

[36] Huhmann M.B., Perez V., Alexander D.D., Thomas D.R. (2013). A self-completed nutrition screening tool for communitydwelling older adults with high reliability: A comparison study. J. Nutr. Health Aging.

[37] Tarqui-Mamani C., Alvarez-Dongo D., Espinoza-Oriundo P., Gomez-Guizado G. (2014). Nutritional status associated with demographic characteristics in older Peruvian adults. Rev. Peru Med. Exp. Salud Publica.

[38] Ahmed I., Kaifi H.M., Tahir H., Javed A. (2023). Malnutrition among patients with Type-2 Diabetes Mellitus. Pak. J. Med. Sci..

[39] Ozkoc M.N.S., Ardic C. (2023). Evaluation of malnutrition frequency and related factors of geriatric patients in need of home healthcare. Rev. Assoc. Med. Bras..

[40] Zhang H., Huang D., Zhang Y., Wang X., Wu J., Hong D. (2023). Global burden of prostate cancer attributable to smoking among males in 204 countries and territories, 1990–2019. BMC Cancer.

[41] Sanz Paris A., Artero A., Burgos Pelaez R., Garcia Almeida J.M., Matia Martin P., Palma Milla S., Zugasti Murillo A., Alfaro Martínez J.J., Calañas Continente A., Chinchetru M.J. (2022). Malnutrition management of hospitalized patients with diabetes/hyperglycemia and hip fracture. Nutr. Hosp..

[42] Davila-Cervantes C.A. (2020). Cardiovascular disease in Mexico 1990–2017: Secondary data analysis from the global burden of disease study. Int. J. Public Health.

[43] Merad-Boudia H.N., Bereksi-Reguig K. (2016). Assessment of Risk of Malnutrition in Elderly Hypertensive Patients with or without Associated Cardiovascular Risk Factors Living at Home (West Algeria) Sidi-Bel-Abbès. Int. J. Clin. Med..

[44] Al Masri F., Muller M., Straka D., Hahn A., Schuchardt J.P. (2022). Nutritional and health status of adult Syrian refugees in the early years of asylum in Germany: A cross-sectional pilot study. BMC Public Health.

[45] Fu Y., Zhu Z., Huang Z., He R., Zhang Y., Li Y., Tan W., Rong S. (2023). Association between Vitamin B and Obesity in Middle-Aged and Older Chinese Adults. Nutrients.

[46] Kaidar-Person O., Person B., Szomstein S., Rosenthal R.J. (2008). Nutritional deficiencies in morbidly obese patients: A new form of malnutrition? Part A: Vitamins. Obes. Surg..

[47] Kaidar-Person O., Person B., Szomstein S., Rosenthal R.J. (2008). Nutritional deficiencies in morbidly obese patients: A new form of malnutrition? Part B: Minerals. Obes. Surg..

[48] Tan B.C., Park Y.S., Won Y., Lee S., Kang S.H., Ahn S.H., Park D.J., Kim H.H. (2021). Preoperative Nutritional Deficiencies in Bariatric Surgery Candidates in Korea. Obes. Surg..

[49] Robinson M.K., Mogensen K.M., Casey J.D., McKane C.K., Moromizato T., Rawn J.D., Christopher K.B. (2015). The relationship among obesity, nutritional status, and mortality in the critically ill. Crit. Care Med..

[50] Ozkaya I., Gurbuz M. (2019). Malnourishment in the overweight and obese elderly. Nutr. Hosp..

[51] Winter J.E., MacInnis R.J., Wattanapenpaiboon N., Nowson C.A. (2014). BMI and all-cause mortality in older adults: A meta-analysis. Am. J. Clin. Nutr..

[52] Chen W., Shi S., Tu J., Liao L., Liao Y., Chen K., Chen L., Huang R. (2022). Nutrition-related diseases and cardiovascular mortality in American society: National health and nutrition examination study, 1999–2006. BMC Public Health.

[53] Wleklik M., Uchmanowicz I., Jankowska-Polanska B., Andreae C., Regulska-Ilow B. (2018). The Role of Nutritional Status in Elderly Patients with Heart Failure. J. Nutr. Health Aging.

[54] Wang X., Cheng Z. (2020). Cross-Sectional Studies: Strengths, Weaknesses, and Recommendations. Chest.

